# Hydrostatic pressure wheel in water distribution systems

**DOI:** 10.1038/s41598-025-20292-3

**Published:** 2025-10-23

**Authors:** Kazem Shahverdi, Ronny Berndtsson

**Affiliations:** 1 Department of Water Science and Engineering, Faculty of Agriculture, Bu-Ali Sina University, Hamedan, 65178-38695 Iran; 2 Centre for Advanced Middle Eastern Studies and Division of Water Resources Engineering, Lund University, Lund, Sweden

**Keywords:** Energy recovery, Genetic algorithm, Hydrostatic pressure wheel (HPW), Optimization, Rotational speed, Water distribution systems

## Abstract

This study presents a transformative advancement in water-energy nexus management by developing a hydrostatic pressure wheel (HPW) system that simultaneously optimizes water level regulation and hydropower generation in open-channel irrigation systems (OCISs)—a dual functionality not achieved by existing technologies. While conventional waterwheels focus solely on energy production, our HPW design leverages hydrostatic pressure dominance to provide precise hydraulic control while extracting renewable energy, addressing two critical needs in irrigation infrastructure with a single integrated solution. The research introduces key innovations beyond current literature: (1) a variable-speed HPW operation strategy that dynamically adjusts to flow conditions, achieving superior performance (45% efficiency, 3.5 kW power output) while maintaining water level deviations below 2.7%—a 40–50% improvement in control accuracy compared to fixed-speed systems and (2) the first coupled numerical framework integrating OCIS hydraulics with HPW dynamics and multi-objective optimization (NSGA-II) to resolve the inherent trade-off between energy maximization and hydraulic stability. The results revealed that variable-speed operation considerably outperforms conventional fixed-speed designs in both energy yield and regulation precision with respectively up to 25% and 60% improvement. These advancements establish HPWs as a new class of smart hydraulic structures that convert traditionally wasteful energy dissipation into renewable power generation while enhancing irrigation management—a capability absent in all prior waterwheel applications documented in literature.

## Introduction

Hydropower is among the most reliable, efficient, clean, and earliest energy resources, and it has played a considerable role in civilization. Evidences date back over 2,000 years to ancient Greece and Egypt, where water wheels were used to grind grain [1]. Despite the limitations and environmental, social, and economic concerns about large dams, small/micro/pico hydropower systems could open new avenues for green and renewable power generation towards more sustainable developments. Open channel irrigation systems (OCISs) have large potential for small/micro/pico hydropower and comprise canal networks that may be managed by hydraulic structures such as gates, weirs, ramps, steps, head drops, and chutes, and some of them may target hydraulic energy dissipation for regulating flow properties such as velocity and depth [2]. However, rather than dissipation, this potential could be employed to generate renewable electricity, particularly as the difference in energy level is often more than 0.5–3 m. This energy can play a significant role in isolated or remote locations with limited or almost no access to the grid or reliable power sources to automate structures near OCISs with kilometers of length. It could introduce a new level of integrated water resources management for developing regions, remote, and less well-served communities, empowering them to more efficiently utilize irrigation systems while enjoying the added values of renewable energy through cost-effective novel solutions, enhancing agricultural performance, by moving towards more sustainable solutions [3].

Various traditional hydropower technologies, such as Francis, Kaplan, Cross-flow, and Pelton, may not be the best option for high-head or high-flow locations, due to damage to aquatic ecology, and not economical and cost-effective for low-head or low-flow locations. Recent advances in novel hydropower technologies can pave the way for harnessing energy from OCISs. Novel and flexible hydropower technologies such as Archimedes screw turbines, also referred to as hydraulic screws, can operate in a wide range of flow rates and head combinations that traditional turbines cannot, while at the same time can be safer for aquatic life, especially fish, and allowing to pass sediments which could reduce disturbing of erosion and sedimentation as well as sediment transportation process [4]. Various studies have focused on applications in low-head and moderate-flow locations, particularly in OCISs [5, 6]. For example, water wheels are example of technology that can pave new avenues for green and renewable hydropower, particularly in water conveyance systems, such as OCISs. Water wheels have a rich historical background dating back to ancient civilizations. They were first developed in ancient Greece and Rome used to lift water, power mills, and machinery during the Middle Ages [7]. Several types of water wheels, including undershot, overshot, breastshot, backshot, and hydrostatic pressure have been developed, each with distinct design characteristics. The first is the simplest and most common, where the water flows beneath the wheel, suitable for low-flow situations with less efficiency than other designs. In the second one, water is fed onto the wheel from above, ideal for utilizing gravity with a significant head of water and is highly efficient. The third has combined features of the first and second ones, on which water flows at mid-height directly, making it versatile and adaptable to different flow rates and water sources. The fourth operates with the water above the wheel hitting the wheel’s back, causing it to rotate [8]. Hydrostatic power wheel (HPW) is a type of water wheel and the simplest hydrostatic pressure converter to translate water energy to mechanical energy.

Vidali et al. [9] conducted experiments and developed methods with dimensional analysis to better understand the factors influencing the water wheel’s operation. A breastshot water wheel was constructed and a series of tests under controlled conditions was conducted. Variables such as water flow rate, wheel speed, and load were systematically varied to collect performance data. The torque, rotational speed, and power output were measured. The study found that the efficiency of the breastshot water wheel is significantly influenced by the flow rate and the wheel’s rotational speed. Optimal performance was achieved at specific combinations of these parameters. The findings highlight the importance of precise control over operational parameters to maximize the efficiency of such water wheels.

The Dethridge wheel for developing power from very low head sites in open channels was studied. To assess the potential of the wheel for electricity generation, a physical model was built and tested. A three-dimensional numerical model of the Dethridge wheel using a commercial Computational Fluid Dynamics (CFD) code Flow-3D was used to show that the rotational speed has a linear relationship with torque and flow rate at constant water levels and that the hydraulic efficiency achieved up to 65% efficiency [10].

The CFD technique was used to evaluate the hydrodynamic characteristics and energy extraction potential of an undershot water wheel turbine by combining advanced turbulence modeling with parametric geometry optimization. The numerical results revealed that optimized blade geometry can achieve a maximum hydraulic efficiency of 42% at specific speed ratios, which represents a 15–20% improvement over conventional undershot designs. Pressure distribution profiles showed that maximum energy extraction occurs when the blades are approximately 30–40% submerged, with deeper submergence leading to increased drag losses [11].

In Alnaqbi et al. [12], the potential of hydropower energy in the Middle East and North Africa region was explored, highlighting that the focus should be on smaller-scale and run-of-river hydropower projects to minimize environmental impacts. Hydropower generation was examined in irrigation water distribution systems in Indonesia using different blade shapes to optimize the efficiency of the Dethridge wheel [13]. A kinetic water wheel was theoretically and experimentally investigated under supercritical flow conditions. The results showed that maximum efficiency is generated under high Froude numbers and a water wheel speed-to-flow velocity ratio of 0.27, led to a maximum efficiency of 72% [14].

To improve energy efficiency, reduce pressure oscillations, and enhance operational performance under dynamic load conditions, an advanced pressure control strategy for hydrostatic transmissions in wheel loaders was proposed by introducing an optimal-pressure control strategy that dynamically adjusts pump displacement and valve settings to maintain near-constant system pressure. The results showed that 10–20% reduction in fuel consumption can be achieved compared to conventional systems [15].

The optimal spacing between multiple water wheel hydrokinetic units in a rectangular canal under mild slope subcritical flow conditions was studied under various unit spacing configurations (ranging from 1.5D to 4D, where D is wheel diameter) on velocity distribution, water surface profiles, and turbulence characteristics. The results demonstrated that spacing below 2.5D causes significant upstream backwater effects (up to 12% head increase) and downstream velocity deficits (reaching 30% reduction), while spacings beyond 3.2D show negligible interference with less than 5% efficiency loss per unit [16].

The influence of operating conditions on breastshot waterwheel performance was studied through combined experimental and numerical approaches. The numerical model, validated against experimental data, revealed that wheel performance peaks at 65–70% efficiency when the upstream water level reached 30–35% of the wheel diameter. The study quantified how excessive submergence beyond 40% diameter led to significant drag losses while insufficient submergence under 20% caused substantial splashing losses, providing crucial design guidelines [17].

A comprehensive review of energy recovery technologies for use in water distribution systems and irrigation networks was performed by assessing the potential of technologies to harness energy from excess pressure, thereby contributing to the water-energy nexus by improving system efficiency and sustainability. The results highlighted pump-as-turbine as a highly cost-effective solution, especially for small to medium-scale applications, whose efficiency ranges from 60 to 80%. However, a major challenge is dropping its efficiency under off-design operating conditions. The Francis turbines are recognized for their high efficiency and suitability for a wide range of flows and heads with efficiencies between 80 and 90% in optimal applications, making them a benchmark for performance in medium-head scenarios. Regarding the Pelton and Kaplan turbines the efficiency ranges from 80 to 90% and from 85 to 90%, respectively, but their complexity can increase costs. The pump-as-turbine cost is the most economical option while conventional Francis, Pelton, and Kaplan turbines involve a significantly higher initial cost due to their custom design and manufacturing for specific site conditions [18].

A detailed case study investigating the technical and economic feasibility of using pumps-as-turbines for energy recovery within a collective irrigation system in Calabria, Italy, was presented by [19] focusing on the specific challenges and opportunities in irrigation networks, which are characterized by high flow rates, seasonal operation, and the necessity for pressure reduction. The analysis revealed a substantial amount of dissipated hydraulic energy that can be a viable resource for renewable power generation, aligning with the water-energy nexus concept [19]. In [20, a methodology for optimal techno-economic design and placement of energy-recovering turbines within water distribution systems was presented by integrating hydraulic model with optimization algorithms to determine the best locations, number of turbines, and their operating setpoints to maximize net present value or energy recovery. A 75% efficiency was presented as a key performance parameter for the proposed solution [20].

HPW, leveraging the kinetic and potential energy of water, is appropriate for low-flow and head locations, effective and efficient in generating electricity, inexpensive, robust, and fish-friendly, sediment continuity, comparable to hydraulic screws, and easy to build and may generate electricity at locations with a head of even less than one meter. In HPW, the blade speed is close to the water velocity; Thus, the entry and exit losses are negligible. The leakage loss is 7–13% at the maximum efficiency. The turbulent losses increase with higher wheel speeds that can be reduced by streamlining the blade to reduce vortex formation. A flexible lip is expected to create such a curvature that increases efficiencies at higher discharges and allows for passage of larger sediment and debris preventing the wheel from clogging [21]. It is appropriate for locations with head differences of 0.2–1.0 m and flow of up to 1.5 m^3^/s and may generate a power of 25 kW/m. The power outputs range from 1.6 to 9.2 kW/m. Note that its theoretical efficiency ranges from 50 to < 100%. In this respect, even small head differences of 0.35 m are effective [22].

An HPW system was designed that integrates theoretical hydraulic modeling with experimental validation in a laboratory flume under various flow conditions of 0.1–0.5 CMS and head differences of 0.3–1.2 m. The results demonstrated the system’s ability to maintain water level regulation within ± 2 cm while simultaneously achieving energy conversion efficiencies of 55–65%. The research revealed that the HPW’s unique blade geometry and pressure distribution characteristics enable stable operation across a 40% variation in flow rates without compromising its regulatory function, while experimental measurements show a power output range of 50–300 W suitable for small-scale energy needs. The study provides design guidelines for implementing these dual-purpose systems in irrigation networks, demonstrating through both modeling and physical testing how the HPW can effectively balance hydraulic control requirements with renewable energy generation in decentralized water management applications [23].

This study addresses a critical gap in the literature by introducing a hydrostatic pressure water wheel (HPW) designed to simultaneously regulate water levels and generate energy in open-channel irrigation systems (OCISs)—a dual functionality never before explored for this technology. The research pioneers three transformative innovations: (1) a novel HPW configuration capable of operating under both variable and constant rotation speeds to optimize water level control and energy production; (2) the integration of this HPW into the Automatic Irrigation Conveyance System Simulation (AICSS) framework, creating the first OCIS simulator to couple hydraulic regulation with energy recovery; and (3) the application of a Multi-Objective Optimization approach (NSGA-II) to reconcile competing objectives—maximizing energy output while minimizing deviations from target water depths—a methodological advance with broad implications for sustainable agriculture. By validating this framework on the E1R1 canal case study, we demonstrate how HPWs can revolutionize irrigation networks, transforming passive water delivery systems into active, energy-generating infrastructure. This work not only expands the theoretical understanding of hydrostatic wheels but also provides a practical blueprint for their implementation in real-world OCISs, where precision water management and renewable energy generation are urgently needed.

## Materials and methods

**Fig. 1:**
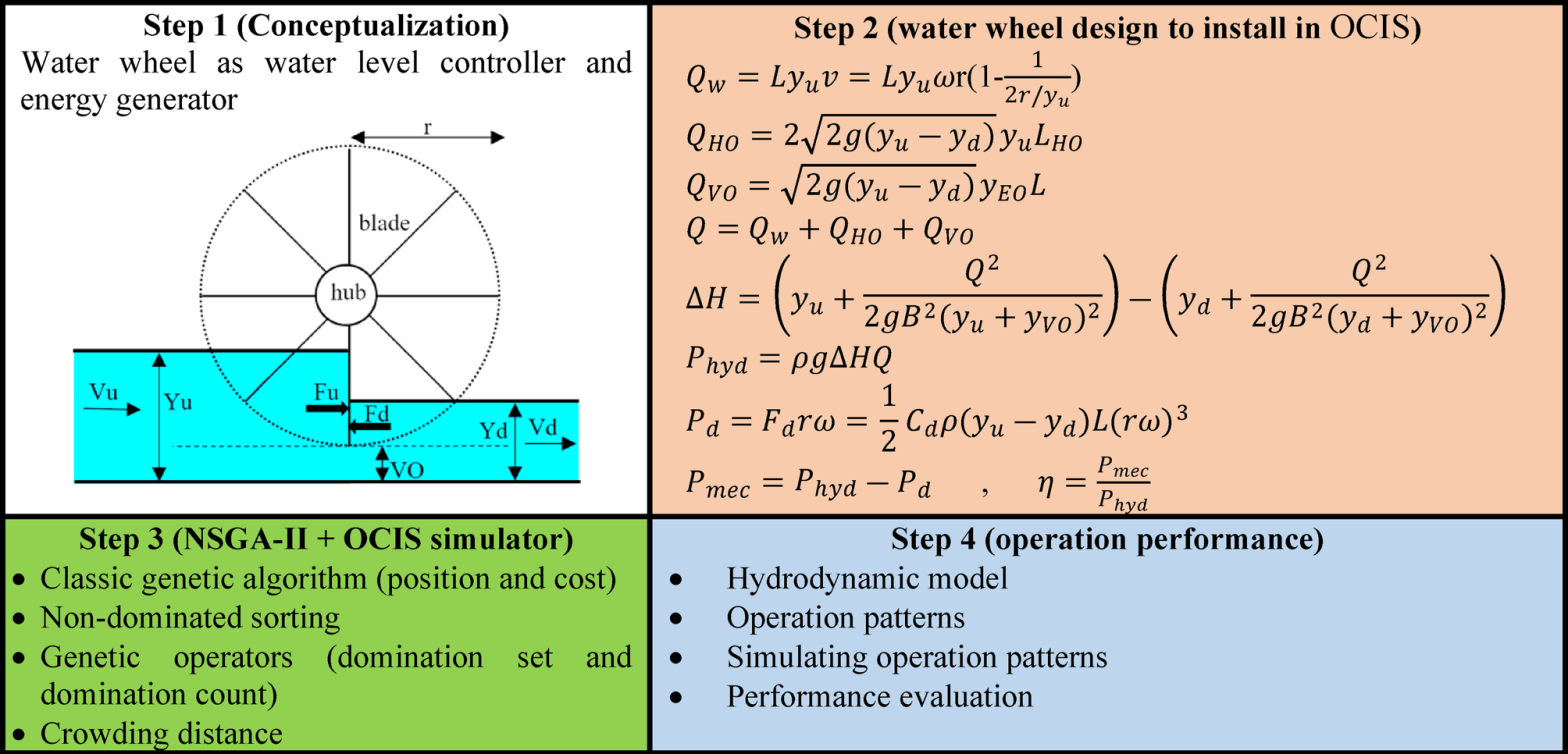
Methodological flowchart for this research in employing water wheels in irrigation water canals.

A methodological flowchart for this study is given in Fig. 1 Step 1 presents the research conceptualization, specifically the installation of HPWs in an OCIS, rather than existing structures, with the hypothesis that HPWs can simultaneously control water levels and generate energy. The geometric and hydraulic parameters, as well as the hydrostatic forces, are illustrated in the figure. In Step 2, the theory of energy generation of HPWs is presented, meaning how much energy can be generated by HPWs based on the relevant parameters involved and theoretical formula. In step 3, the procedure of designing an optimal and accessible HPW is shown, targeting generating maximum mechanical energy and controlling water level precisely. Single-objective optimization algorithms such as genetic algorithms are not applicable in this study, hence, multi-objective optimization algorithm of NSGA-II was used. NSGA-II was chosen due to its wide utilization and simple implementation in comparison to other multi-objective algorithms. It specifies the ranks and sorts of solutions using non-dominated sorting, reduces computational complexity, and uses a selectivity approach. Automatic irrigation conveyance system simulation (AICSS) was used to simulate the case-studied OCIS. In Step 4, the operation of OCIS was examined using performance criteria.

### Theory, principles, and formulation

**Fig. 2:**
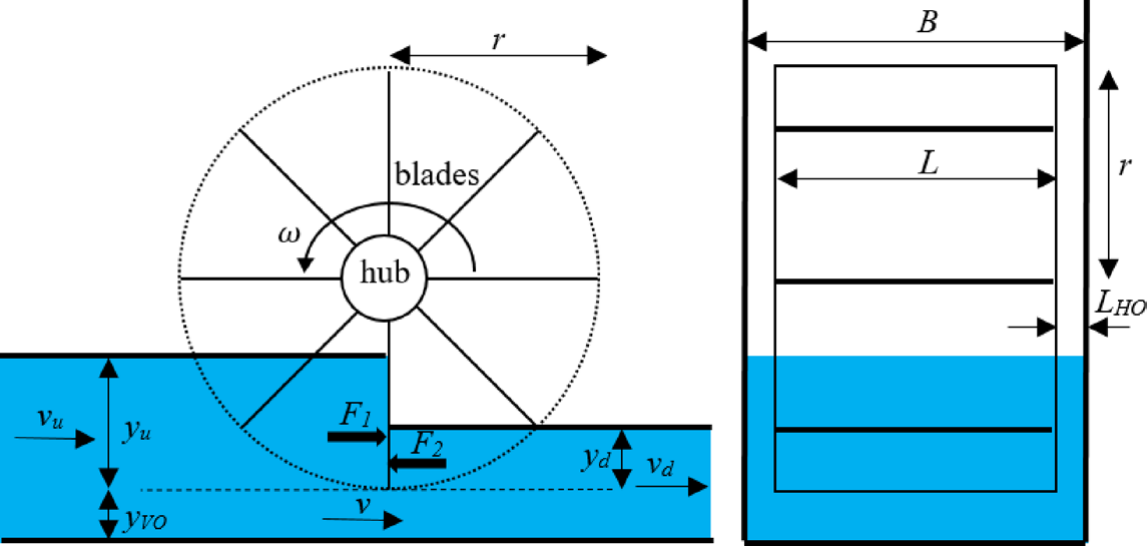
Longitudinal and cross-sectional schematic views and relevant parameters of HPW.

The longitudinal and cross-sectional schematic views and relevant parameters of HPW are presented in Fig. 2, where *B* is the canal width, $r$ is the HPW radius, ${\text{and} y}_{u}$ and ${y}_{d}$ are respectively the upstream and downstream water depths related to the lower edge of the blade, ${v}_{u}$ and ${v}_{d}$ are respectively the upstream and downstream water velocities related to the lower edge of the blade, $v$ is the velocity of flow passing under the lower edge of the blade, $\omega$ is the rotation speed of the wheel, and $L$ is the HPW width. A vertical opening is the distance between the canal bed and the lower edge of a vertical blade. Depth related to the vertical opening is equal to the opening denoted by ${y}_{VO}$. Likewise, the horizontal opening refers to the horizontal distance between the canal side wall and the blade, whose length is denoted by ${L}_{HO}$. The vertical and horizontal openings contribute to flow leakage, calculated using the Torricelli equation, as $v=\sqrt{2g{(y}_{u}+{y}_{VO})}$ under free flow conditions. Since the HPW blade numbers, the HPW radius, and the HPW width are the preliminary parameters of an HPW, selecting their appropriate values is essential.

The flow through the main body of HPW (${Q}_{w}$) is determined using the continuity equation, assuming that the flow velocity equals the blade speed. In the continuity equation, the flow velocity is multiplied by the flow area (Eq. 1). It is important to note that the flow area of the main body of HPW, which generates mechanical torque, is $L\times {y}_{u}$:

1 $$\begin{aligned} Q_{w} & = L \times y_{u} \times v = L \times y_{u} \times \left( {\frac{1}{Y}\mathop \smallint \limits_{r}^{{r - y_{u} }} y \times \omega \times dy} \right) \\ & = L \times y_{u \times } \omega \times {\text{r}} \times \left( {1 - \frac{1}{{2r/y_{u} }}} \right) \\ \end{aligned}$$

The leakage flow (${Q}_{l}$), the primary cause of energy losses, is the sum of the leakages from the vertical and horizontal openings computed respectively by:

2 $${Q}_{VO}=\sqrt{2g\left({y}_{u}-{y}_{d}\right)}{\times y}_{EO}\times L$$

3 $${Q}_{HO}=2\sqrt{2g\left({y}_{u}-{y}_{d}\right)}{\times y}_{u\times }{L}_{HO}$$

4 $${Q}_{l}={Q}_{HO}+{Q}_{VO}$$

where ${Q}_{VO}$ represents the leakage from vertical openings, ${Q}_{HO}$ denotes leakage from horizontal opening, and ${y}_{EO}$ indicates water depth relevant to the equivalent opening, with its value depending on the radial position of the blades; therefore, an average value obtained through calibration was utilized. The total flow, $Q$, in the canal, which comprises ${Q}_{w}$ and ${Q}_{l}$, is calculated according to:

5 $$\begin{aligned} Q & = L \times y_{u \times } \omega \times {\text{r}} \times \left( {1 - \frac{1}{{2r/y_{u} }}} \right) \\ & \quad + \sqrt {2g\left( {y_{u} - y_{d} } \right)} \times y_{EO} \times L \\ & \quad + 2\sqrt {2g\left( {y_{u} - y_{d} } \right)} \times y_{u} \times L_{HO} \\ \end{aligned}$$

As shown in Eq. (5), all parameters except for ${y}_{EO}$ and $\omega$ are known; therefore, ${y}_{EO}$ can be found by trial and error if $\omega$ is known so that the obtained flow using Eq. (5) becomes equal to the real flow introduced to the canal.

The weight of the water contained on the upstream side of the blades is considered the cause of HPW rotation. The difference between upstream and downstream water heads is referred to as the net head. The resulting forces must be balanced to develop the theoretical model of HPWs; however, the pressure force upstream exceeds that downstream. The Bernoulli’s principle is employed to calculate the net hydraulic head $\Delta H$ as:

6 $$\Delta H = \left( {y_{u} + \frac{{Q^{2} }}{{2gB^{2} \left( {y_{u} + y_{VO} } \right)^{2} }}} \right) - \left( {y_{d} + \frac{{Q^{2} }}{{2gB^{2} \left( {y_{d} + y_{VO} } \right)^{2} }}} \right)$$

In Eq. (6), the first parenthesis denotes the summation of the pressure and velocity heads upstream of HPW, while the second parenthesis indicates the summation of the pressure and velocity heads downstream of HPW. The hydraulic power of ${P}_{hyd}$ is calculated by:

7 $${P}_{hyd}=\rho \times g\times \Delta H\times Q$$

where $\rho$ represents water density and $g$ denotes gravitational acceleration. The mechanical power (P) generated by the system can be expressed as the difference between the hydraulic power and the drag power (${P}_{d}$), as shown in Eq. (8). The drag power component is determined by the product of drag torque and the HPW’s rotational speed. The drag torque itself is derived from the drag force (${F}_{d}$) acting on the wheel multiplied by the radius of the HPW (Eq. 9):

8 $${P={P}_{hyd}-P}_{d}$$

9 $${P}_{d}={(F}_{d}r)\omega =\frac{1}{2}{C}_{d}\rho A{v}^{2}r\omega =\frac{1}{2}{C}_{d}\rho \left({y}_{u}-{y}_{d}\right)L{\left(r\omega \right)}^{2}r\omega =\frac{1}{2}{C}_{d}\rho ({y}_{u}-{y}_{d})L{(r\omega )}^{3}$$

where ${C}_{d}$ is the drag coefficient, set at 0.4 in this research based on the findings of [23]. They experimentally measured the drag power, flow rate, rotation speed, upstream water depth, and downstream water depth and incorporating these variables into Eq. (8) to determine the drag coefficient. Note that the upper limit of rotation speed is derived from ${F}_{\omega }=\frac{r\omega }{\sqrt{g{y}_{u}}}<1$ (where ${y}_{u}<r$). Finally, the efficiency $\eta$ is calculated by:

10 $$\eta =\frac{P}{{P}_{hyd}}$$

The theoretical model outlined above offers two significant advantages: a) leakage and turbulence effects are incorporated in Eq. (5), and b) water depth and rotation speed are dependent variables; thus, upstream water level can be adjusted alongside energy generation.

### Simulator

AICSS is an advanced version of the irrigation conveyance simulation system (ICSS) and open-source software developed by Shahverdi [24] to model water flow in rivers and channels, used as an OCIS simulator in this research. It can simulate time- and space-variant flows with numerous hydraulic structures. To model OCISs and calculate velocity and depth at computational nodes, the following equation sets (continuity and momentum equations) are solved in an iteration process:

11 $$\partial A/\partial t+\partial Q/\partial x=0$$

12 $$\partial Q/\partial t+\partial ({Q}^{2}/A)/\partial x+gA(\partial y/\partial x-{S}_{0}+{S}_{f})=0$$

where ${S}_{0}$: OCIS’s slope, ${S}_{f}$: friction slope determined by Manning’s equation, $t$: time, $y$: water depth at computational nodes, and $x$: length. Note that ICSS, and consequently AICSS, was validated using experimental data, gathered in a concrete rectangular flume with 1.25 m wide, and 210 m long, 0.0019 longitudinal slope, and 0.012 Manning’s roughness. The validation results showed 0.29%, 0.68%, and 1.9% error in predicting upstream peak depth, downstream peak depth, and peak flow, respectively [25]. The AICSS of the E1R1 OCIS was provided in this research using geometric and hydraulic data and validated using real data available in the literature.

### Optimizer

NSGA-II is a well-known, common, robust, and popular algorithm for solving multi-objective optimization problems and stands for non-dominated sorting genetic algorithm II. It is an extended version of the original NSGA algorithm that solves problems with multiple conflicting objectives. Note that in a classic genetic algorithm, two factors of position and cost are explored in the defined population set to find the optimal value. The multi-objective NSGA-II optimization algorithm uses the classic genetic algorithm with its operators, such as crossover (recombining genetic material from parents) and mutation (introducing small random changes) along with non-dominated sorting and crowding distance. It evolves a population of candidate solutions and identifies the dominant relationships among them, enabling it to efficiently produce a diverse array of high-quality solutions, referred to as the Pareto front, which illustrates the trade-offs between multiple objectives.

NSGA-II’s principle is grounded in elitism and diversity preservation. The elitist principle ensures that the best-performing individuals, often referred to as elites, are carried over to the next generation, allowing effective solutions to be retained and not lost. To uphold a variety of solutions, diversity preservation employs a technique known as crowding distance, which measures how dispersed solutions are within the objective function space. It ranks solutions based on their objective function values and subsequently calculates the distance between each solution and its two nearest neighbors. The larger the crowding distance, the more valuable the solution, resulting in higher priority and greater diversity. The final set of optimal solutions on the Pareto front strikes a good balance among different objectives, where improvement in one objective may lead to a decline in another.

Consider an initial population $P$ of size $n$, represented as $P={(X}_{1}, {X}_{2},{\dots , X}_{n})$, where ${X}_{i}$ denotes solutions represented by decision variables. The objective function, ${f}_{k}\left({X}_{i}\right)={(f}_{1}\left({X}_{i}\right),{f}_{2}\left({X}_{i}\right), \dots {f}_{m}({X}_{i}))$, is evaluated for each solution, as a minimization target, subjected to ${g}_{z}\le 0, z=1,\dots ,q,$ where $q$ is conditions and $m$ indicates objectives. In this research, six objective functions ($m=6$) were defined, three of which maximize mechanical energy efficiency, defined by Eq. (10), for three HPWs of 1–3 and three others minimize water depth deviations from associated target value upstream of HPWs of 1–3, defined by root mean square error (RMSE), defined by:

13 $$RMSE=\sqrt{\frac{\sum_{t=1}^{T}{({y}_{u}^{t}-s)}^{2}}{T/t}}$$

where ${y}_{u}^{t}$ is the upstream water depth in each time interval $t$, $s$ is the target depth, $and T$ is the total simulation time. Having three regulating gates and replacing them with suitable HPWs, three $RMSE$ s was calculated. Thus, the objective functions were defined as Eqns. (14)-(19). The overall objective function was defined as the sum of all distinct objective functions as (Eq. 20):

14 $$minimize {f}_{1}\left({X}_{i}\right)=minimize {RMSE}_{1}$$

15 $$minimize {f}_{2}\left({X}_{i}\right)=minimize {RMSE}_{2}$$

16 $$minimize {f}_{3}\left({X}_{i}\right)={minimize RMSE}_{3}$$

17 $$maxmize {f}_{4}\left({X}_{i}\right)=maxmize {\eta }_{1}=minimize (-{\eta }_{1})$$

18 $$minimize {f}_{5}\left({X}_{i}\right)=maxmize {\eta }_{2}=minimize (-{\eta }_{2})$$

19 $$minimize {f}_{6}\left({X}_{i}\right)=maxmize {\eta }_{3}=minimize (-{\eta }_{2})$$

20 $$minimize f\left( {X_{i} } \right) = minimize\,\left( {RMSE_{1} + RMSE_{2} + RMSE_{3} - \eta_{1} - \eta_{2} - \eta_{3} } \right)$$

The modified canal width of ${B}_{c}$ of pools 1–3 and three HPW radiuses of *r* that will be installed in pools 1–3 were defined as decision variables (total six decision variables), subject to $1.5\le r\le 2.0 m and 0.1\le {B}_{c}\le B$.

After evaluating the objective function for each solution $${X}_{i}$$, they are sorted into diverse frons according to the dominance definition, based on which solution $${X}_{i}$$ dominates solution $${X}_{j}$$ if $${f}_{k}\left({X}_{i}\right)\le {f}_{k}\left({X}_{j}\right) for all k and {f}_{k}\left({X}_{i}\right)<{f}_{l}\left({X}_{j}\right) for at least one l$$. Next, fronts $${F}_{1},{F}_{2}, \dots$$ are generated, where $${F}_{1}$$ contains non-dominated solutions, $${F}_{2}$$ includes solutions dominated by one solution in $${F}_{1}$$, and so forth. In the next step, the crowding distance of $${d}_{i}={d}_{i}+({f}_{k}\left({X}_{i+1}\right)-{f}_{k}\left({X}_{i+1}\right))/({f}_{k}^{max}-{f}_{k}^{min})$$ is calculated for each solution $${X}_{i}$$ by sorting the solutions in the front $${F}_{n}$$, where $${f}_{k}^{max}$$ is the maximum value of objective $$k$$ and $${f}_{k}^{min}$$ are the minimum one in the sorted front. Then, the solution in the superior front is chosen when two solutions belong to different fronts, while if two solutions are on the same front, the one with a better crowding distance is chosen. Subsequently, crossover and mutation are applied, and finally, a new population is formed by combining parents and offspring. This process is repeated multiple times to achieve the desired criteria.

In this research, the NSGA-II algorithm was implemented in the MATLAB platform and integrated with the AICSS hydraulic model to optimize the defined objectives. The developed algorithm in this research is as follows.

Start
2. Initialize the population with random candidate solutions
Simulate the OCIS with AICSS and evaluate the objective functions for each candidate solution
Perform non-dominated sorting to rank the solutions based on dominance
Assign crowding distances to the solutions to maintain diversity.
Create the offspring population through selection, crossover, and mutation operators.
Evaluate the objective functions for the offspring population.
Combine the parent and offspring populations.
Check if the termination condition is met: If yes, go to step 10; otherwise, go to 4
Return the final Pareto front as the set of non-dominated solutions
End.

### Operational cases

**Fig. 3:**
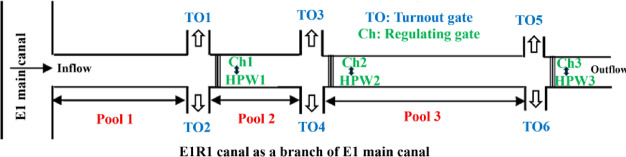
The E1R1 case study canal in this research.

The E1R1 OCIS selected in this research is a typical medium-scale irrigation system that has hydraulic conditions compatible with HPW operation (Fig. 3), whose specifications is presented in Table 1. The upstream water depth (${y}_{u}=1.2$ m) was held constant across all pools, according to the canal’s operation policy to maintain turnout flow predictability. The canal width ($B$) is 1.5 m converted to 1.0 m in downstream to accommodate flow partitioning at turnouts, while the downstream depths (${y}_{d}$) were derived from uniform flow calculations under the given side slope (S = 1.5) and flow range (Q = 0.8–1.2 CMS). It this canal, HPWs can operate efficiently across varying discharges [26]. It is important to clarify that the ± 20% variation in flow rate does not represent short-term fluctuations (hourly or daily) but rather reflects seasonal changes over the crop cultivation period. For instance, flow rates in spring are typically higher than in summer. While the flow remains relatively stable on a daily or even weekly basis, it may vary between different periods of the season. This seasonal variation is accounted for in our analysis, rather than random measurement uncertainty. Please also note that while the canal has a trapezoidal shape, it transitions to a rectangular cross-section before the turbine installation. Therefore, the wheel was installed in the rectangular section, not the trapezoidal one.

**Table 1:** Specifications of the studied canal used in this research for pool 1–3.

	Unit	Definition	Pool 1	Pool 2	Pool 3
$B$	m	Canal width	1.5	1.0	1.0
${y}_{u}$	m	Upstream water depth	1.2	1.2	1.2
${y}_{d}$	m	Downstream water depth	0.86	0.57	0.37
S	–	Side slope	1.5	1.5	1.5
Q	CMS	Flow	0.8–1.2	0.8–1.2	0.8–1.2

This study evaluated two operational strategies for suitable HPWs based on constant and variable rotational speeds control. In the constant speed case, the HPWs maintain fixed rotational speeds regardless of flow variations, causing the upstream water depth to fluctuate with changes in canal discharge (Q). This represents a departure from conventional canal operation policies that require constant upstream water depths, potentially affecting turnout performance. However, this study quantifies the magnitude of these depth variations to assess their practical implications. While this approach offers cost advantages by eliminating the need for speed control systems, it comes with reduced operational flexibility and potential hydraulic instability.

In contrast, the variable speed case adjusts HPW rotational speeds proportionally with flow rates to maintain constant upstream water depths, thereby preserving the original canal operation policy. This ensures that turnout flows remain solely dependent on gate openings, mirroring traditional system behavior. Although it provides superior hydraulic stability and operational compatibility with existing infrastructure, more sophisticated control mechanisms and higher implementation costs are required. The comparison of these two operational modes offers valuable insights for system designers: the constant speed HPW presents an economical solution for systems where minor depth variations are acceptable, while the variable speed HPW is better suited for applications requiring strict depth control, such as precision irrigation networks.

Maximum absolute error (MAE) and integral absolute error (IAE) along with RMSE were used to evaluate the developed model performance defined respectively as [27]:

21 $$MAE=\frac{\text{max} (\left|{y}_{u}^{t}-s\right|)}{s}$$

22 $$IAE=\frac{\frac{t}{T} \sum_{t=0}^{T}(\left|{y}_{u}^{t}-s\right|)}{s}$$

where $s$ is the setpoints (target depths) that is 1.2 m for all pools. Zero is ideal for these indices.

## Results and discussion

### Optimal parameters

To find the optimal parameters of an HPW that may be installed in the OCIS, NSGA-II and AICSS were coupled and used in this research. The coupled models can simulate the canal and carry out optimization simultaneously. The optimal parameters of NSGA-II are presented in Table 2.

**Table 2:** Optimal values obtained for NSGA-II.

Parameter	Value
Crowding distance	0.857
Crossover rate	0.7
Mutation rate	0.05
Maximum iteration	300
Initial population size	25

**Fig. 4:**
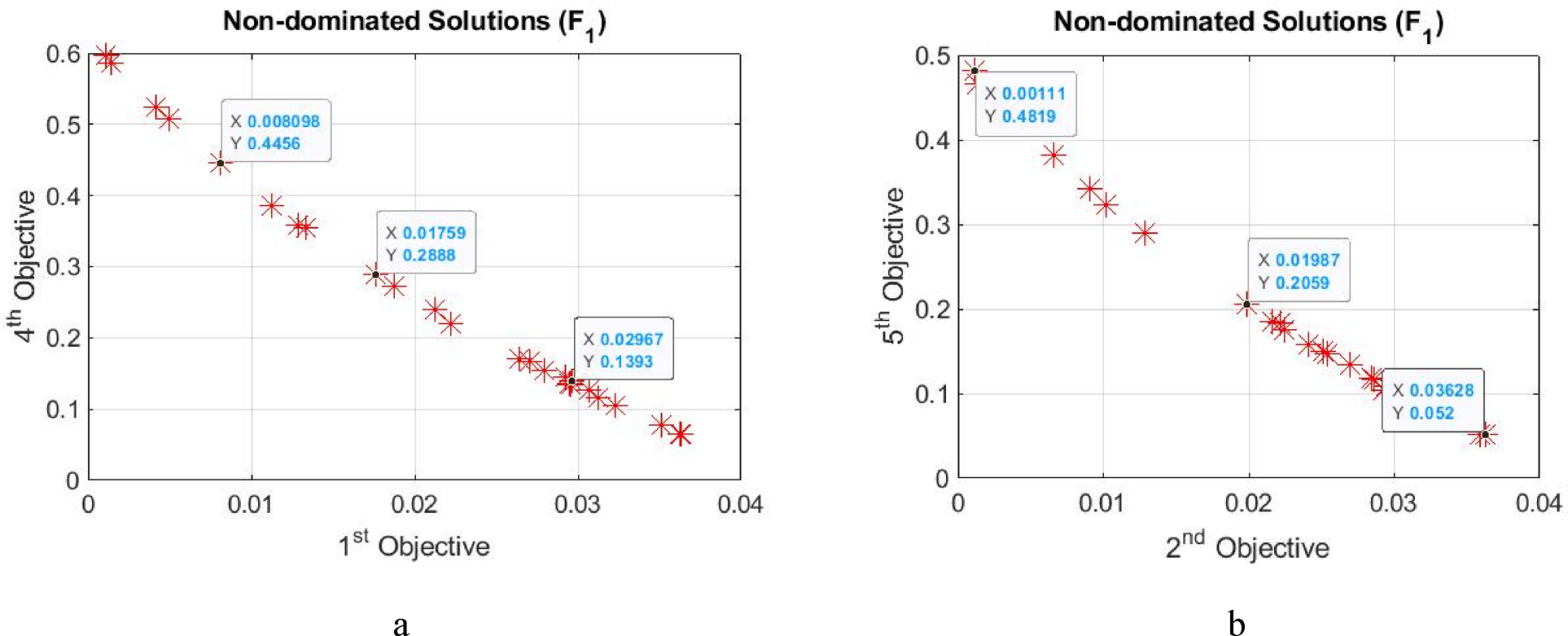
The Pareto front for, (**a**) objectives 1 and 4 in pool 1, and (**b**) objectives and 5 in pool 2.

The maximum crowding distance was found 0.857, suggesting a well-distributed set of solutions along the Pareto front preventing clustering and ensuring diversity in the optimized parameters. In the first pool, the lower and higher crowding distances during the optimization process were 0.295 and 0.768, respectively. The solution with a higher crowding distance was chosen, whose RMSE and efficiency were 0.008 and 0.445, respectively, maintaining diversity among solutions, measuring how close a solution is to its neighbors in the objective space, determining which individuals will be retained during selection, aiming at preserving diverse solutions while converging toward the Pareto front. Overall, higher crowding distances are desirable. In pool 2, the lower and higher crowding distances were 0.288 and 0.857, respectively. The point with the higher crowding distance is the optimal solution again, whose RMSE and efficiency were 0.019 and 0.205, respectively. The Pareto front for two objectives, as two examples, is presented in Fig. 4a and b, respectively. Note that the crowding distance is a measure of diversity preservation in the Pareto front. A value closer to 1 ensures better spread of solutions and balances exploration and exploitation.

The specifications of two random points in the Pareto front, for example in Fig. 4a, are as follows. The first one has an RMSE of 0.017 and an efficiency of 0.288 with a crowding distance of 0.546 and the other one has an RMSE of 0.029 and an efficiency of 0.139 with a crowding distance of 0.295. The RMSE and efficiency of the best solution respectively are 0.008 and 0.4456, showing a minimum deviation of water depth from the desired value and maximum efficiency produced.

The crossover rate was obtained at 0.7 which guarantees sufficient exploration in the search space by promoting the exchange of gens between parent solutions, providing a robust balance between introducing new genetic material and preserving high-quality solutions. The crossover value is consistent with common recommendations in genetic algorithms and NSGA-II literature, where crossover rates typically range between 0.6 and 0.9. The relatively low mutation rate of 0.05 chosen maintains stability by introducing controlled perturbations to avoid premature convergence. It introduces slight perturbations without disrupting the convergence process. The mutation rates are kept small to maintain stability while allowing for occasional exploration of new regions in the search space. The choice of 300 iterations, determined at 300 by trial and error, was based on convergence analysis. We observed that the algorithm achieved satisfactory Pareto-optimal solutions within this range, with further iterations yielding negligible improvements. Computational efficiency was also a consideration. The initial population size of 25 was adequate to initiate the search process, though a larger population might have further enhanced exploration in the early stages. A smaller population size was selected to reduce computational overhead while ensuring sufficient diversity. This was validated through sensitivity analysis, where larger sizes did not significantly improve solution quality but increased runtime. The canal and HPW optimal parameters were determined using coupled models of NSGA-II and AICSS (Table 3).

**Table 3:** Obtained optimal parameters.

	unit	Definition	Pool 1	Pool 2	Pool 3
${B}_{c}$	m	Modified canal width	0.60	0.407	0.494
$r$	m	HPW radius	1.975	1.975	1.975
$L$	m	HPW width	0.560	0.407	0.454
$N$	–	Blade number	8	8	8
${y}_{VO}$	m	Depth of vertical opening	0.025	0.025	0.025
${y}_{HO}$	m	Depth of horizontal opening	0.02	0.02	0.02
${y}_{EO}$	m	Water depth relevant to equivalent opening	0.075	0.075	0.075
$\omega$	rad/s	Rotation speed	0.832	0.922	0.895

The upstream and downstream depths of each regulating gate are given in Table 1. The head difference between the upstream and downstream of each regulating gate is available energy in unit weight that rotates the wheel in an HPW system. As shown, the upstream depths are all 1.2 m, and the downstream depths are 0.86, 0.57, and 0.37 m, respectively, for the regulating gates 1–3. Since regulating gate 2 is a dropped gate with a drop height of 1 m, the downstream depth doesn’t have any effects on the available energy.

The optimal values of the HPW radiuses 1–3 were all obtained at 1.975 m, and those of canal width were obtained at 0.6 m, 0.407 m, and 0.494 m, respectively. Applying the horizontal openings of 0.02 m, the water HPW widths 1–3 were obtained at 0.56 m, 0.367 m, and 0.454 m, respectively. Subsequently, the maximum values of the rotation speed of the HPWs 1–3 were calculated as 1.707 rad/s, 1.708 rad/s, and 1.712 rad/s, respectively, by employing $\frac{r\omega }{\sqrt{g{Y}_{u}}}<1$. However, the optimal rotation speeds were finally determined at 0.832, 0.922, and 0.895 rad/s, respectively. Parameters $N=8$, ${y}_{VO}=0.025$ m, ${y}_{HO}=0.02$ m, and ${y}_{EO}=0.075$ m were chosen for all regulating gates based on the suggestions given in the literature [23].

### Constant rotation speed, variable upstream depth

As obtained and mentioned earlier for the design flow of 1 CMS, the rotation speeds of HPWs 1–3, in case the water depth upstream of all structures is 1.2 m and the water depth downstream of structures 1–3 are 0.86, 0.57, and 0.37 m, are 0.832, 0.922, and 0.895 rad/s, respectively, determined based on the specifications in Table 3 and Eq. 5. The respective output powers are 1492 W, 2495 W, and 2495 W with efficiencies of 45%, 21%, and 31%, which are in line with Cassan, Dellinger [23]. Note that a maximum efficiency of 72% was obtained using a kinetic turbine [14]. As can be seen, the last two output powers are the same while there is no influencing water depth downstream of HPW 2, which can be explained by the fact that the flow leakage is greater in HPW 2 since there is no hydrostatic pressure downstream that acts as a resistance force. Due to the flow leakage, the efficiency is about 1.5 times less in HPW 2 compared to HPW 3.

The deployment of various regulatory devices, such as sluice gates is required for the canal automation for effective management of water, which is intricate, particularly in remote areas where most canals are situated. Integrating an HPW that automatically regulates water levels by adjusting its rotational speed is one potential solution since it would produce energy that could be used to power the sensors and control systems necessary for monitoring and managing water flow and data transmission. In addition, any excess energy generated could be utilized to support the local electricity grid in these secluded locations. The global average of the per capita electricity consumption by each household subscriber is 182 kWh/month. The monthly power generated using HPWs 1–3 is about 18,700 kWh that could be sold to household subscribers.

**Fig. 5:**
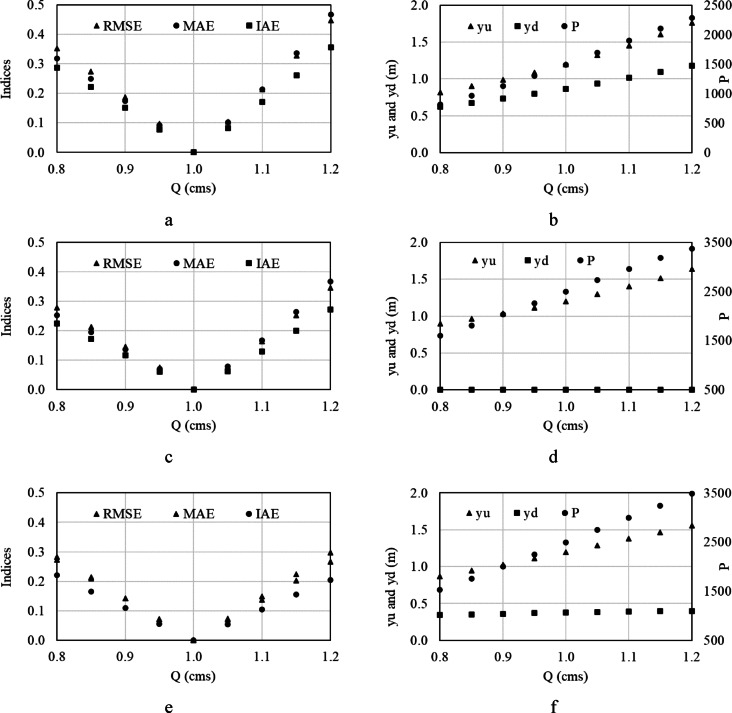
Performance indices, upstream, and downstream water depth variations, and power generated with the designed HPWs 1–3 for different flow conditions. The rotation speed is constant.

As mentioned, the canal flow range is 0.8–1.2 CMS that was applied to the canal employing HPW and the indices were calculated and examined. Figure 5a and b show findings in the first pool, Fig. 5c and d show findings in the second pool, and Fig. 5e and f show findings in the third pool, as the rotation speed is constant and upstream water depth is variable. As seen, the water depths and output powers are less as flow is less than design flow and those are more as flow is more than design flow. In both cases, the indices are greater due to the more water deviation from the target value.

MAE and IAE are less than 20% when the flow variations are ± 10% of the design flow, meaning flow is 0.9–1.1 CMS. However, MAE and IAE are a bit much, and the performance is acceptable. MAE reaches up to 45%, 37%, and 30% in the first, second, and third pools, respectively, when the flow variations are ± 20% of the design flow (0.8–1.2 CMS), leading to a poor performance for the first pool and a fair performance for the second and third pools. The fair performance is between poor and good performance. Similarly, IAE reaches up to 36%, 26%, and 20% in the first, second, and third pools, respectively, when the flow variations are ± 20% of the design flow, and the same performance and justification as MAE can be explained. The minimum output powers for HPWs of 1–3 are 822, 1602, and 1528 W, and the maximum output powers for HPWs of 1–3 are 2285, 3375, and 3480 W, respectively. This can be compared to a similar study where a similar power generation was reported [28].

In a nutshell, the OCIS operation is not affected when the flow variations are around the design flow. It becomes worthwhile as the flow is much more or less than the design flow. Notwithstanding the foregoing, constant rotation speed is an advantage economically since the cost of constant rotation speed turbines is less than that of variable rotation speed turbines. This can be a research topic to be investigated in future research.

### Variable rotation speed, constant upstream depth

**Fig. 6:**
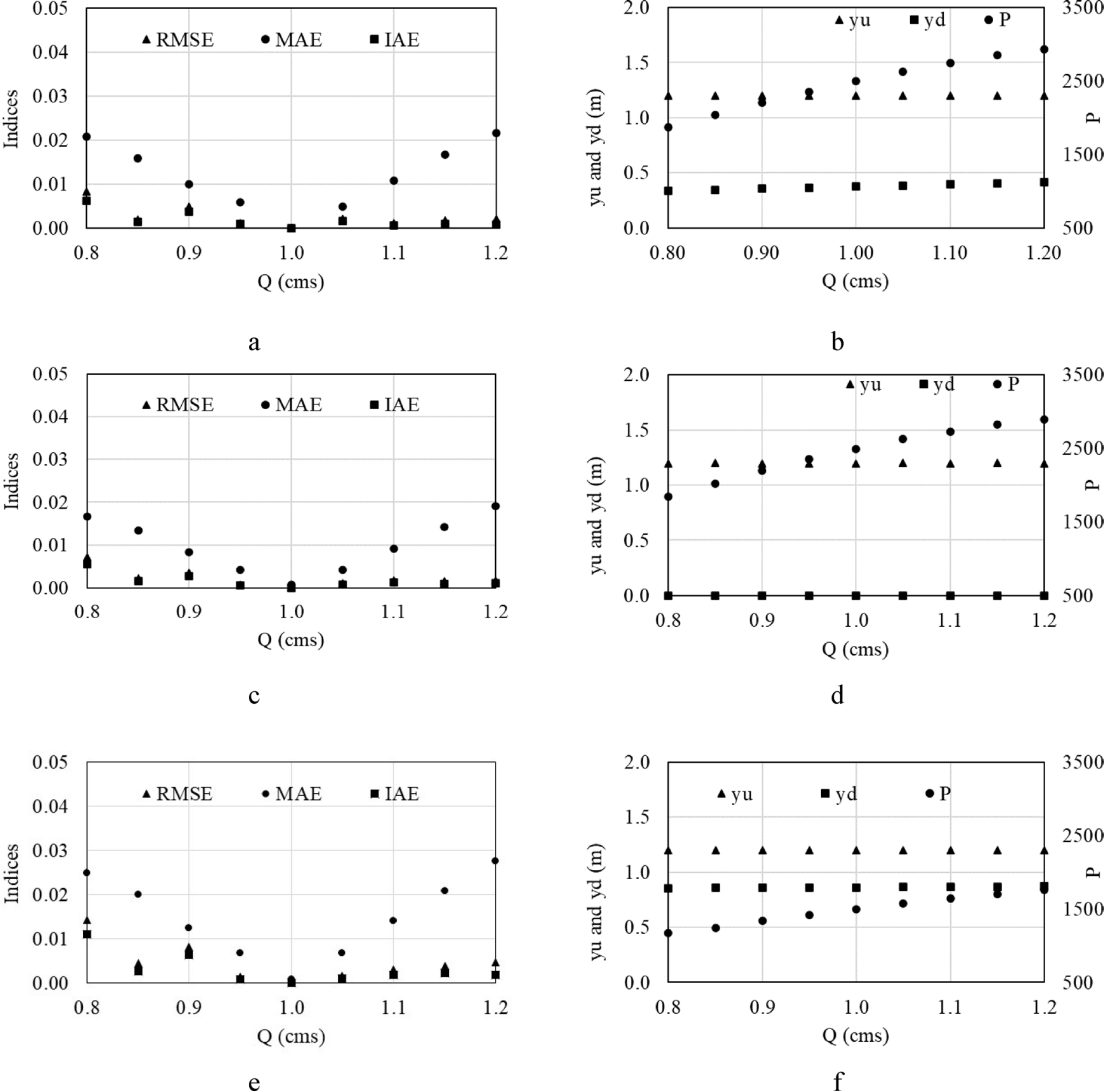
Performance indices, upstream, and downstream water depth variations, and power generated with the designed HPW 1 (**a** and **b**), 2 (**c** and **d**), and 3 (**e** and **f**) in different flow conditions. The rotation speed is variable.

In this case, the rotation speed increases with increasing the flow proportionally and vice versa. This case was defined to have nearly constant water depth upstream of HPWs, causing the OCIS to operate precisely with high performance. In this case, the current operation of OCIS is affected very little although the cost of a variable rotation speed turbine increases. The rotation speed is determined in such a way that the water depth remains in the target depth as much as possible. Based on the results, the water depths downstream of HPW remain nearly constant for all flow conditions investigated due to the OCIS slope and less backwater effect of the downstream end depth of each pool on the upstream end depth (Fig. 6b, d, and f).

In comparison to the previous case, the range of output power value is relatively less. Regarding HPW 1 for example, the output power is between 1170 and 1758 W for variable rotation speed (average output power is 1521 W) and between 822 and 2285 W for constant rotation speed (average output power is 1481 W) while flow range from 0.8 CMS to 1.2 CMS. The average output power is approximately the same. Here, the leakage flow is nearly constant for any flow conditions since the upstream and downstream water depths are constant. For lower flows, the output power of the constant rotation speed case is lower since the head and head difference is lower. For higher flow, the output power of the constant rotation speed case is also higher due to the same reason. However, the flow leakage also increases as the head difference increases. Comparing the constant and variable rotation speed results, it can be concluded that the performance is approximately the same in terms of generated power.

In contrast to the output power, the OCIS performance is better in terms of the operation in case of variable rotation speed. As shown in Fig. 6a, c, and e, the maximum MAE is 2.7% and the maximum IAE is 1.1% in this case is much less than those in the constant rotation speed case, which were 45% and 36%, respectively. The low MAE and IAE values show that the water depth variations in OCIS are small so that the turnouts located upstream of HPWs receive precise constant flow. Since the flow delivered to the turnouts is directly dependent on the water depth, the less the water depth deviation from the target value, the more accurate the receiving flow. With a constant water depth, the flow delivering to the turnouts can be regulated by just its gate opening so that the operation becomes easy.

The performance of constant rotation speed and variable rotation speed can be compared in different aspects of sensitivity to flow variations, power generation characteristics, and economic and operational trade-offs. Regarding the first aspect, the system with constant rotation speed exhibits significant sensitivity to flow changes. At ± 10% deviation from the design flow (0.9–1.1 CMS), MAE and IAE remain below 20%, indicating acceptable performance. However, at ± 20% deviation (0.8–1.2 CMS), MAE and IAE rise sharply (up to 45% and 36%, respectively, in the first pool), degrading water depth regulation. This configuration maintains exceptional stability with variable rotation speed, with MAE and IAE consistently below 2.7% and 1.1%, respectively, across all flow variations. The adaptive speed adjustment ensures precise water depth control, even under large flow fluctuations. Regarding power generation, constant rotation speed HPW generates comparable average power (e.g., 1481 W for HPW 1) to the variable-speed case but with a wider output range (e.g., 822–2285 W for HPW 1). Peak power output is higher (up to 3480 W for HPW 3) due to increased head differences at extreme flows. The variable rotation speed HPW delivers a narrower power range (e.g., 1170–1758 W for HPW 1) with similar average output (1521 W for HPW 1). Power generation is more stable but lacks the extreme peaks observed in the constant-speed case. Regarding the third aspect, constant rotation speed HPW economically advantageous due to lower turbine costs, making it suitable for systems with predictable, small flow variations (± 10%) where minor depth deviations are acceptable. This is Ideal for cost-sensitive deployments where energy generation is prioritized over precision. While variable rotation speed HPW involves higher initial costs it ensures superior operational reliability, particularly in canals with large or unpredictable flow variations (± 20% or more). This is essential for applications requiring strict water-level control, such as automated turnout systems where flow accuracy is critical.

**Fig. 7:**
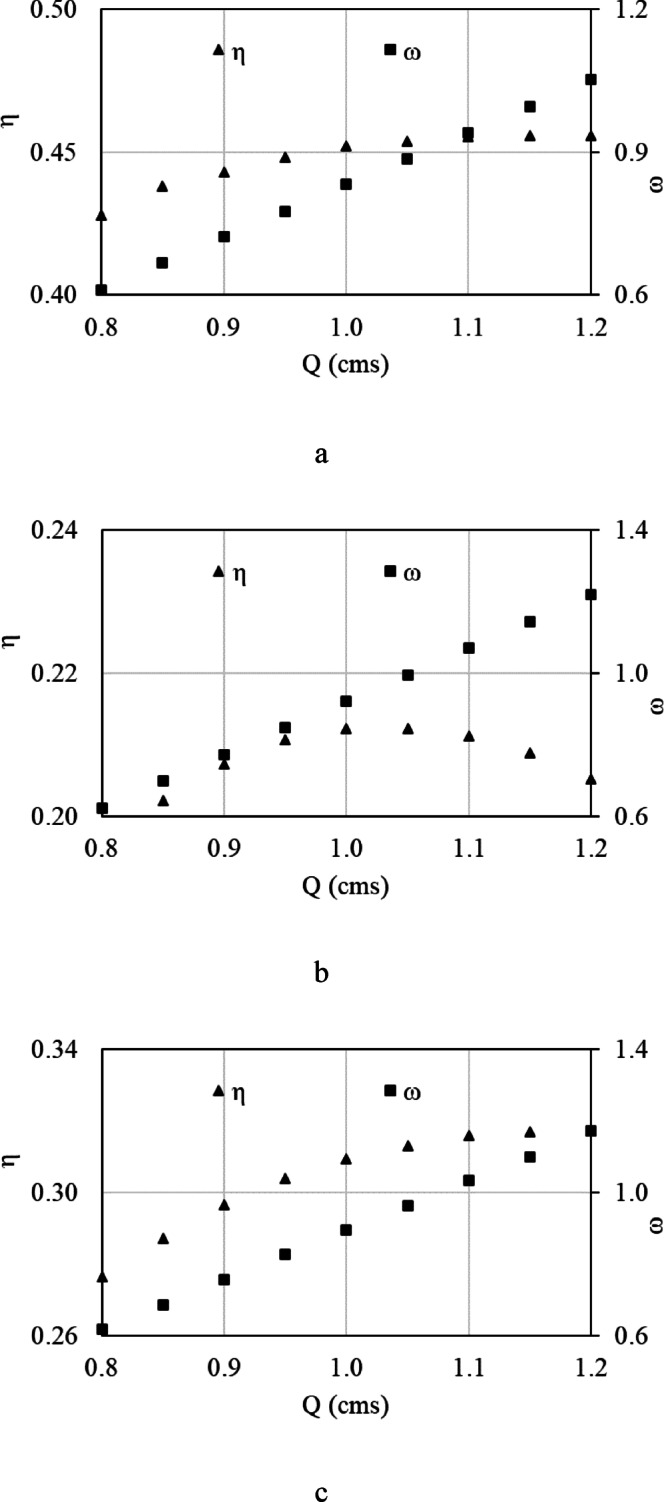
The rotation speed and efficiency variations with the variation of flow rate for HPWs 1–3.

The rotation speed and efficiency variations with the variation of flow rate is presented in Fig. 7a–c for HPWs of 1–3, respectively. As explained earlier, the rotation speed increases with increasing flow rate, which in turn causes the output power to increase. The efficiency increases as the flow rate increases until a maximum in efficiency is reached. Then, the flow rate increase causes an efficiency reduction. It should be noted that the output power in such a case is more important than the efficiency since the output power is in the order of 1000–2000 W, which is enough for local utilization of such a control system.

A preliminary economic assessment was conducted using the key parameters for HPW3 with a 2.495 kW mechanical power output, generating an estimated 7186 kWh of electrical energy annually considering 4 months of operation. The financial viability was evaluated using a simple payback period model, based on an initial investment of $6238, an electricity price of $0.17/kWh, and annual operational expenses (cash outflow) of $374. The calculated annual net cash flow was $1036, leading to an estimated simple payback period of 6.5 years. Although it indicates a strong return on investment, we assumed that most civil works in the canal could be excluded due to existing drops in the canal, and the total costs comprise electrical and mechanical component costs.

## Conclusions

In the present study, the NSGA-II optimizer and AICSS simulator were coupled to design an optimal HPW to be replaced instead of regulating gates. Water depth deviations from respective target values and efficiencies in pools 1–3 were six objective functions. The HPW radiuses and canal widths in pools 1–3 were the decision variables. Two cases of constant rotation speed and variable rotation speed were examined. The results indicate that the algorithm successfully converged toward a set of Pareto-optimal solutions, balancing multiple competing objectives maximizing efficiency and minimizing water depth deviations from target value.

The results showed that the optimal HPW radius 1–3 was 1.975 m, and the optimal canal width was 0.6, 0.407, and 0.494 m, respectively, for pool 1–3 leading to the HPW widths 0.56, 0.367, and 0.454 m. The higher crowding distance was 0.768 in pool 1, whose RMSE and efficiency were 0.008 and 0.445, respectively, meaning the optimal efficiency was 44.5%. In pool 2, the higher crowding distance was 0.857 with the RMSE and efficiency of 0.019 and 0.205. The output powers were 1492, 2495, and 2495 W for pools 1–3, respectively. MAE reaches up to 45%, 37%, and 30% in pools 1–3 with constant rotation speed, respectively, leading to a poor performance in pool 1 and a fair performance in pools 2 and 3. The maximum MAE was 2.7% in the case of variable rotation speed.

The minimum output powers for HPWs of 1–3 were 822, 1602, and 1528 W, and the maximum output powers were 2285, 3375, and 3480 W, respectively, for the case with constant rotation speed. Comparing the constant and variable rotation speed results, it can be concluded that the performance is the same in terms of generated power without remarkable differences. The OCIS operation with variable rotation speed was better than that with constant rotation speed, thereby, it is recommended.

According to the results, it can be concluded that the HPW with rotation speed is a good choice for exploiting energy from OCIS with low available hydraulic power while maintaining water depth at respective target value. This coupling (NSGA-II with AICSS) enables simultaneous optimization of hydropower generation and water-level control, addressing both energy and irrigation needs in a single system. This dual functionality surpasses conventional gates, which lack energy recovery capabilities. The variable rotation speed configuration demonstrates exceptional resilience to flow fluctuations (± 20% of design flow), maintaining stable water depths (MAE ≤ 2.7%) while ensuring consistent power output (1.2–3.5 kW). The NSGA-II algorithm successfully resolves competing objectives (efficiency vs. depth deviation), yielding Pareto-optimal designs with crowding distances up to 0.857. This ensures a balanced trade-off between energy exploitation and hydraulic precision. Despite of strength and effectiveness, there are some weaknesses and limitations as well. While variable rotation speed excels in control precision, its higher turbine costs and complexity may limit adoption in budget-constrained projects. The constant-speed alternative, though economical, suffers degraded performance (MAE up to 45%) under large flow deviations. The optimal HPW radii (∼1.975 m) and canal widths (0.4–0.6 m) are site-specific. Scaling to larger or smaller canals may require re-optimization, increasing computational effort. Despite competitive efficiency (45%), the output power (≤ 3.5 kW) remains suitable only for small-scale applications. Larger grids would require parallel HPW installations, raising space and maintenance concerns.

A modular design architecture where HPW units are deployed in parallel or series to match specific site conditions is suggested. A tandem or multi-stage configuration could be developed to handle higher total head drops by distributing the pressure reduction across several wheels, making the technology applicable to a wider range of topographies beyond the very low-head applications initially studied. Another significant future expansion involves transforming the HPW from a passive energy recovery device into an active smart grid component. By incorporating a controlled brake system on the axle, the rotational speed of the wheel could be finely regulated. This would allow the HPW to function as an adjustable pressure reduction valve, providing precise downstream pressure control in response to real-time demand signals from a supervisory control and data acquisition (SCADA) system, thereby simultaneously managing network pressure and optimizing energy production.

## Data Availability

Data sets generated during the current study are available from the corresponding author (Kazem Shahverdi, E-mail: [k.shahverdi@basu.ac.ir] (mailto:k.shahverdi@basu.ac.ir)) on reasonable request.

## List of symbols

AICSS: Automatic irrigation conveyance simulation system
CFD: Computational fluid dynamics
Ch: Check structure
HPW: Hydrostatic power wheel
IAE: Integral absolute error
ICSS: Irrigation conveyance simulation system
MAE: Maximum absolute error
NSGA: Non-dominated sorting genetic algorithm
OCIS: Open channel irrigation system
RMSE: Root mean square error
TO: Turnout
*B*: Canal width
$${C}_{d}$$: Drag coefficient
CMS: Cubic meter per second
$${F}_{d}$$: Drag force
$$g:$$: Gravitational acceleration
$$L$$: HPW width
${L}_{HO}$: Horizontal distance between the canal side wall and the blade
$$n$$: Population size
*P*: Mechanical power
$$P$$: Initial population
$${P}_{d}$$: Drag power
$${P}_{hyd}$$: Hydraulic power
$$Q$$: Total flow
${Q}_{HO}$: Leakage from horizontal opening
${Q}_{VO}$: Leakage from vertical openings
${Q}_{w}$: Flow through the main body of HPW
$${Q}_{l}:$$: Leakage flow
$$r$$: HPW radius
$${S}_{0}$$: Canal slope
$${S}_{f}$$: Friction slope
$$x$$: Length
$${X}_{i}$$: Solution *i*
$$t$$: Time
$v$: Velocity of flow passing under the lower edge of the blade
${v}_{d}$: Upstream water velocity related to the lower edge of the blade
${v}_{u}$: Downstream water velocity related to the lower edge of the blade
$$y$$: Water depth
${y}_{d}$: Downstream water depth related to the lower edge of the blade
${y}_{u}$: Upstream water depth related to the lower edge of the blade
$${y}_{VO}$$: Vertical opening
$\Delta H$: Net hydraulic head
$$\rho$$: Water density
$\omega$: Rotation speed of wheel
$$\eta$$: Efficiency
